# A novel aspiration technique to assess cervical remodelling in patients with or without cervical shortening: Sequence of first changes, definition of cut-off values and impact of cervical pessary, stratified for cervical length

**DOI:** 10.1371/journal.pone.0283944

**Published:** 2023-04-06

**Authors:** Ioannis Kyvernitakis, Philien Lauer, Marcel Malan, Sabrina Badir, Holger Maul

**Affiliations:** 1 Department of Obstetrics and Prenatal Medicine, Asklepios Klinik Barmbek, Asklepios Medical School, Hamburg, Germany; 2 Pregnolia AG, Schlieren, Switzerland; University of Palermo: Universita degli Studi di Palermo, ITALY

## Abstract

**Background:**

The therapeutic significance of the cervical pessary has been confirmed by several studies. However, the underlying mechanism by which pessaries reduce the risk of a preterm birth remains elusive. The aim of this study is to investigate the hypothesis whether the application of a cervical pessary may stabilize the ectocervical stiffness in order to achieve a cervical arrest.

**Methods:**

This is a prospective, controlled, non-interventional, post-market, monocentric, longitudinal, cohort study in a tertiary maternity hospital to determine ectocervical stiffness and its changes measured before and after placement of a pessary in singleton pregnancies with cervical shortening in the mid trimester. In order to assess reference values for cervical stiffness, we measured also singleton pregnancies with normal cervical length in the same gestational week spectrum. The cervical stiffness measured with the Pregnolia System as the Cervical Stiffness Index (CSI, in mbar) shall be the primary endpoint, whilst patient delivery data (gestational age, mode of delivery and complications) will be the secondary endpoint. In this pilot study, up to 142 subjects will be enrolled to have a total of 120 subjects (estimated dropout rate of 15%) to complete the study; pessary cohort: 60 (up to 71 recruited), control group: 60 (up to 71 recruited).

**Discussion:**

Our hypothesis is that patients with cervical shortening will present with lower CSI values and that pessary placement will be able to stabilize the CSI values through further prevention of cervical remodelling. The measurement of controls with normal cervical length shall serve as a reference.

## Introduction

Preterm birth (PTB) has been consistently implicated in a wide range of health medical conditions affecting neonates and contributed in up to more than a half of overall perinatal mortality [1]. Spontaneous PTB (sPTB) has multiple causes. Different risk factors influence the aetiologies leading to a final pathway that accumulates into the precocious cervical ripening. Known risk factors include maternal factors, such as ethnicity, age, social or economic status as well as patient’s history, such a previous preterm birth [2]. Furthermore, pregnancy characteristics, such as infections [3, 4], multiple gestation, biological and genetic markers play a predominant role in this context. However, the precise underlying mechanisms resulting in a sPTB remain unexplained [5].

The cervical pessary is a circular shaped device with a smaller circumference to enclose the cervix and a wider circumference to fix the device within the vagina of different height in the shape of a dome. The effectiveness of pessary therapy in singleton and twin pregnancies with cervical shortening has been underlined in several trials, especially when it is applied earlier than 20 weeks of gestations and when Instructions For Use (IFU) have been considered during placement and follow-up [6–13].

Several plausible therapeutic mechanisms of action have been proposed. First, one hypothesis describes the mechanical-rotating effect of the pessary on the uterocervical angle resulting in a smaller angle which prevents direct pressure on the membranes covering the internal os of the uterus and cervix itself [14, 15]. Second, the mechanical engagement of the cervix is enforced due to the constraining modality of the device [16]. The mucus plug creates an immunological barrier between the chorioamniotic membrane and the decidua [17]. Although studies have shown beneficial effect of cervical pessary in reducing a preterm birth, the reasons why pessaries might fail to prevent it remain unknown.

Identifying risk factors for sPTB through a detailed obstetric and medical history, gynaecological examination and objective tests such as transvaginal ultrasound measurement of cervical length and biomarkers like fetal fibronectin are crucial for PTB management [18]. An assessment of a cervical softness is an important, however so far very subjective part of an obstetrical clinical examination. Similarly, the Bishop score considers the estimated cervicovaginal angle and is–though being influenced by a high inter- and intraobserver variability–a clinically established method for labour pre-induction, but not for prediction of sPTB [19, 20].

Up to date, there is no clinically established diagnostic tool allowing reliable and reproducible assessment of cervical stiffness in pregnant women [20]. Certain attempts to appraise cervical stiffness with a cervical elastography have been conducted [21]. A novel aspiration technique-based device quantitatively assessing this is the Pregnolia System. Cervical stiffness alterations are reflected by the Cervical Stiffness Index (CSI) [22]. The device quantifies cervical stiffness by measuring the amount of vacuum required to deform cervical tissue. The device was already safely used in more than 1000 women. The device is known to be a safe tool to assess cervical remodelling by the determination of cervical stiffness in a quantitative and objective manner. However, there are no works up to date assessing the impact of the cervical pessary application on cervical stiffness.

This study aims to evaluate whether women with cervical shortening indicated for pessary treatment have *a priori* lower CSI values than controls with normal cervical length. The research also aims to evaluate the impact of the cervical pessary treatment on the measured cervical stiffness, and whether cervical stiffness, or its changes correlate with birth outcome.

## Materials and methods

### Study design

A prospective, non-interventional, post-market, monocentric, longitudinal, cohort study will be held in a tertiary maternity teaching hospital to determine ectocervical stiffness and its changes measured before and after the placement of cervical pessary. Patients with singleton pregnancies and cervical shortening below the 3. percentile according to the reference values of Salomon et al. will be recruited in the pessary cohort. The findings will be correlated with the birth outcome. The study schedule is depicted in Table 1.

**Table 1:** Study schedule.

Study Assessment	Information	Screening	Baseline	Follow-up	Postpartum
Visit		1	1	2	Medical Record
Time	Before baseline visit	18^+0^–24^+6^	18^+0^–24^+6^	4 weeks after baseline	4–8 weeks after delivery or termination of the pregnancy
Send / Give Patient Information and Informed Consent Form	X				
Informed Consent process and signature		X			
In- /Exclusion Criteria		X			
Speculum to confirm eligibility			X		
Demographics			X		
Obtain surgical, medical, gynaecological and obstetrical history			X		
Listing of concomitant medication			X	X	X
CL and uterocervical angle measurement			X	X	
CSI measurements			X	X	
Discomfort assessment for CSI and pessary placement			X	X only for CSI	
Adverse Events			X	X	X
Collection of information about preterm birth intervention, delivery and related complications, maternal / fetal health, health economics data					X

A gestational age-matched control of pregnancies with no risk factors for spontaneous preterm birth and normal cervical length will be used for comparison of measured cervical stiffness and outcomes. A total of 142 subjects fulfilling the eligibility criteria will be enrolled. Subjects will be recruited to participate in the study between 18^+0^ to 24^+6^ weeks gestation.

Cervical stiffness is measured with a novel CE-marked medical device, the Pregnolia System, developed by Pregnolia AG and based on research conducted by the Swiss Federal Institute of Technology Zurich (ETH) and University Hospital Zurich (USZ) [22].

Participants will continue to receive all routine obstetrical care and indicated interventions. If the woman agrees to participate in the study, additional study assessments will be conducted during the first visit (18^+0^–24^+6^ weeks of gestation) and the follow-up visit (4 weeks later). Procedures at each visit are detailed and explained in Table 2. The participants will be enrolled during the 18^+0^–24^+6^ week of pregnancy and will be in the study until birth or termination of the pregnancy. The pessaries will be removed at 37^+0^ weeks of gestation according to the national guideline for PTB prevention.

**Table 2:** Procedures at each visit.

Visit #1 at 18^+0^–24^+6^ weeks of gestation
• Confirm inclusion/exclusion criteria are met • Inform consent signature and enrolment to the study • Speculum examination to confirm eligibility (after screening & enrolment) • Obtain demographic data • Obtain surgical, medical, gynaecological and obstetrical history • 3 consecutive TVUS for cervical length and uterocervical angle assessment • 3 consecutive CSI measurements with the Pregnolia System • Discomfort assessment of CSI measurement (for both cohorts) and of pessary placement (for pessary cohort only) • Adverse Events
**Follow-up visit (4 weeks later)**
• 3 consecutive TVUS for cervical length and uterocervical angle assessment • 3 consecutive CSI measurements with the Pregnolia System • Discomfort assessment of CSI measurement • Adverse Events
**In absence of participant–post-partum**
• Collection of interventions and/or treatments during pregnancy to prevent preterm birth, if any • Collection of delivery information (onset of labour, mode of delivery, GA at delivery, date of delivery) and newborn data (e.g. weight) • Collection of health status data of mother (hospitalization (yes/no), indication for hospitalization, GA at admission and GA/date at discharge) and neonatal morbidities. • Collection of health economics (date admission to NICU of the baby, date of discharge from NICU of the baby, discharge from hospital (baby)) • Adverse Events

### Study approval and registration

The ethics committee (Ärztekammer Hamburg, project number 2021-100680-BO-ff) has approved the study (study identifier #4011, protocol version 1.0 of June 9^th^, 2021), and the study has been registered on clinicaltrials.gov (NCT05267717). It is intended that the results of the study will be published as a scientific work. Any modifications to the protocol which may impact on the conduct of the study, potential benefit of the subject or may affect subject safety, including changes of study objectives, study design, patient population, sample sizes, study procedures, or significant administrative aspects will require a formal amendment to the protocol.

### Study population

The study population will consist of pregnant women attending the Asklepios Klinik Barmbek, Hamburg Germany at 18^+0^–24^+6^ gestational weeks for the second trimester scanning. Study eligibility will be determined at the first visit and women can be selected for the study if they meet the inclusion and exclusion criteria described below. Written informed consent is required from all patients included in this study in order to have their data from medical records used in this research project.

Our research will be a longitudinal cohort-study. Hereby, we are planning two cohorts to allow a comparison of the cervical stiffness between the groups: the pessary-cohort consisting of patients with cervical shortening and the control group consisting of patients with normal cervical length (non-pessary cohort).

#### Sample size calculation

In this pilot study, up to 142 subjects will be enrolled to have a total of 120 subjects (estimated dropout rate of 15%) for the study; pessary cohort: 60 (up to 71 recruited), non-pessary cohort: 60 (up to 71 recruited). Currently, it is difficult to perform a formal power analysis since the Pregnolia System is a novel device and there exist only limited clinical data.

#### Inclusion and exclusion criteria

All participants should meet the following inclusion criteria in order to be eligible:

- Pregnant woman recruited between 18^+0^–24^+6^ weeks of gestation
- Maternal age ≥ 18 years
- Subject able to sign approved consent form to participate in the study
- Singleton gestation

Moreover, there are specific inclusion criteria for both the pessary and the non-pessary cohorts. For the pessary cohort, these consist of cervical shortening confirmed by transvaginal ultrasound (TVUS) (CL < 3^rd^ percentile at gestational age at measurement) according to Salomon at al. (14, 32), whilst for the control (non-pessary) group we will include asymptomatic pregnant women with no risk factors for spontaneous preterm birth and with a normal cervical length.

Any possible exclusion criteria will be evaluated for eligibility for the study on the first visit. Exclusion criteria for both pessary and control group will be:

- evidence of fetal anomaly or fetal chromosomal abnormality from fetal scanning
- uterine malformations
- history of diethylstilboestrol (DES) use, (cases of so-called DES daughters, who were exposed to DES *in utero*)
- cervical cerclage or pessary currently in place
- silicone allergy
- painful regular contractions
- abnormal placentation (previa, accreta)
- rupture of membranes
- cervical dilation
- any visible, symptomatic cervical or vaginal infections (this excludes treated, asymptomatic infections)
- known HIV infection
- cervical carcinoma
- the presence on the cervix at the 12 o’clock position of any of the following conditions: Nabothian cyst, cervical myomas, cervical condylomas, cervical endometriosis, cervical tears, scar tissue, cervical ectopy, cervical scarring due to prior LLETZ, cervical squamous intraepithelial lesion, cervical dysplasia, cone biopsy,
- vaginal bleeding evident on exam.

#### Criteria for withdrawal

The subjects will be advised in the Informed Consent Form that they have the right to withdraw from the study at any time without prejudice and may be withdrawn at the Investigator’s discretion at any time when this is considered to be in the interest of the subject. In the case that a subject drops out of the study or is withdrawn, the withdrawal / study termination page in a Case Report Form (CRF) shall be completed. On the withdrawal page of the CRF, the investigator should record the date of the withdrawal, the person who initiated withdrawal and the reason for the withdrawal. Reasonable effort should be made to contact any subject lost to follow up during the study in order to complete assessments and retrieve any outstanding data.

Withdrawal can be decided by the investigator due to an adverse event, a major protocol deviation that may lead to endangerment of the subject, a major protocol deviation concerning the primary outcome related to data quality, an investigator’s concern regarding subject’s health and withdrawal of informed consent. Discontinuation due to an Adverse Event should be documented in the CRFs.

### Study device and indication

The Pregnolia System is used to provide information about the mechanical properties of the uterine cervix by assessing the tissue stiffness through a proxy parameter (the Cervical Stiffness Index, in mbar). The system consists of a reusable component, the Pregnolia Control Unit and a disposable probe, the Pregnolia Probe.

The device is intended to be used in addition to other standard examinations and does not substitute them. Cervical stiffness can be assessed during routine examinations—along with other parameters, such as cervical length measured by ultrasound—in order to gather a supportive data for diagnostics in the field of obstetrics and gynaecology, in particular about cervical remodelling.

### Study device description

**Fig 1:**
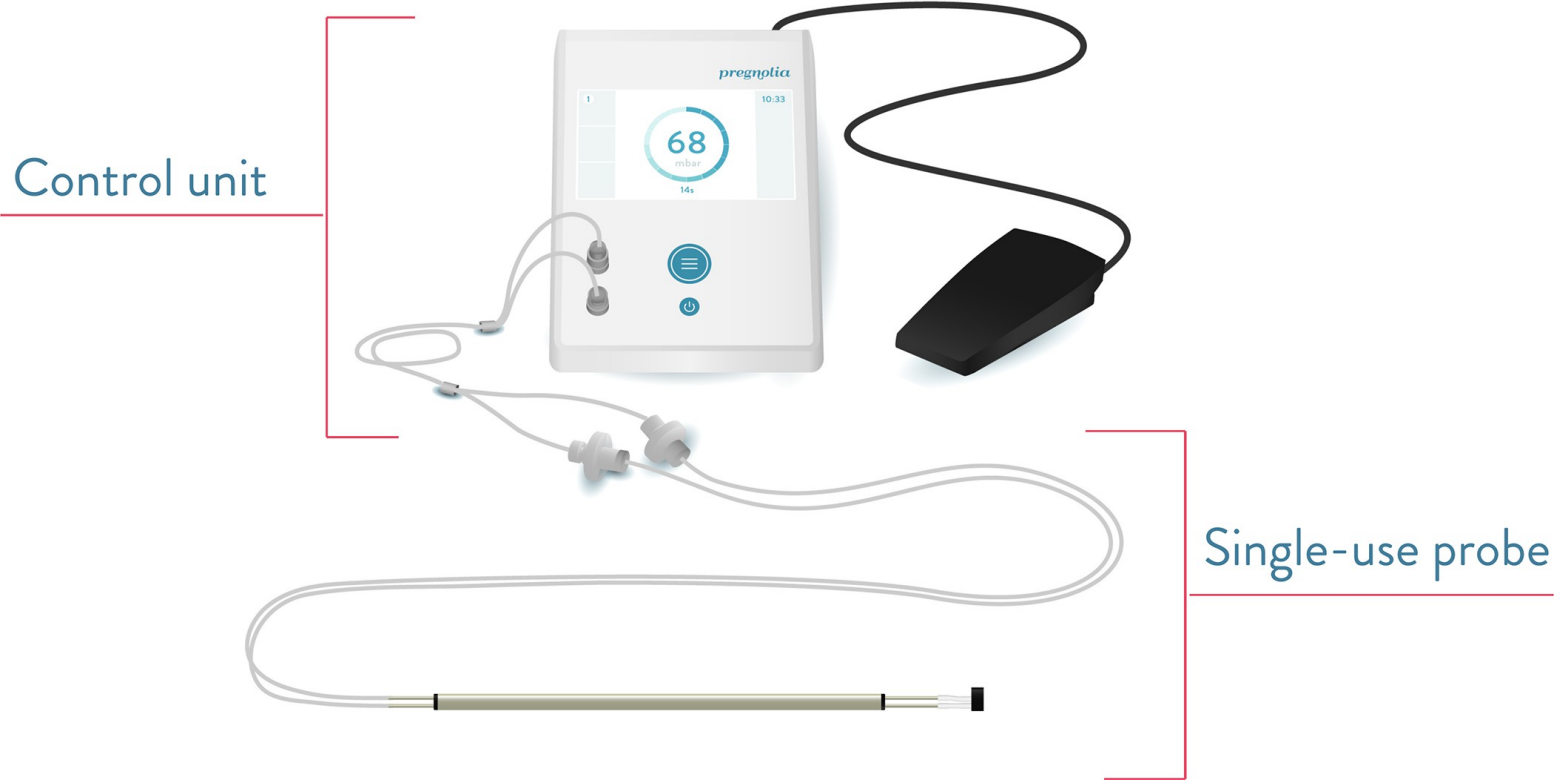
System components: Control unit including control unit console, power supply (not shown), foot switch, connector cable and single-use probe. From the Instructions for Use of the Pregnolia System (www.pregnolia.com/instructions).

**Fig 2:**
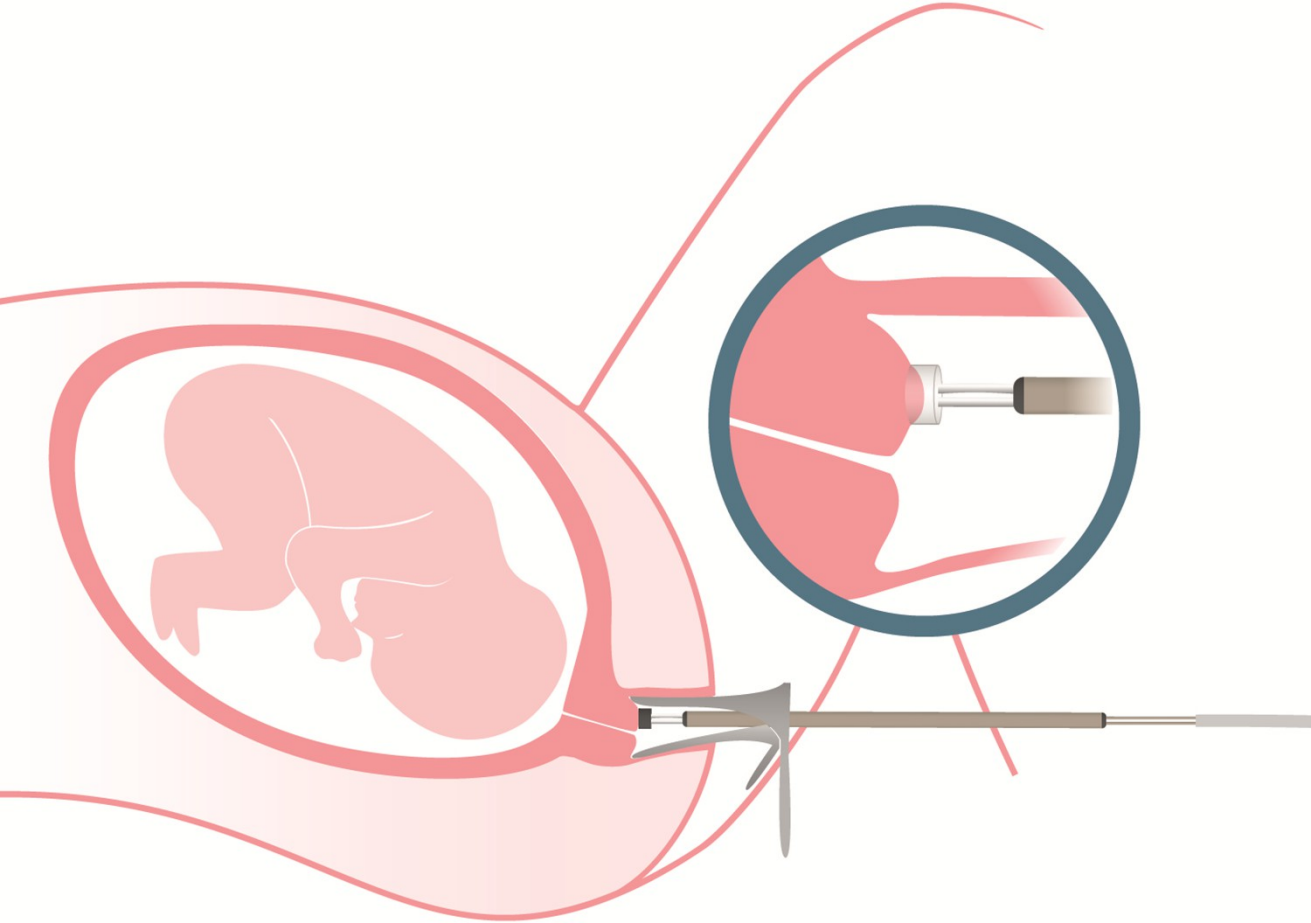
General overview of the method and application. The probe is manually placed on the cervix during a routine gynaecological evaluation, with the aid of a speculum. Adapted from [20].

The Pregnolia System is composed of two products: an active device (Pregnolia Control Unit) and a single-use sterile probe. The Pregnolia system is depicted in Fig 1. The control unit console is an active device with a power supply, foot switch, connector cable and an integrated pump that generates vacuum. The single-use sterile probe is connected to the control unit console through a connector cable. Air filters on the probe prevent microbiological contamination of the control unit. The probe is transvaginally applied on the anterior lip of the cervix with the aid of a speculum and an external light source. A general overview of the system application is shown in Fig 2.

To determine the tissue stiffness, the control unit console generates a weak vacuum, and the cervical tissue is slowly deformed into the probe tip. The vacuum level required to displace the tissue into the probe tip by a fixed distance (4 mm) characterizes the tissue stiffness; it is defined as the Cervical Stiffness Index (CSI) and expressed in mbar. The stiffer the tissue, the higher the closing pressure.

#### Risks / Benefits

The study will be performed on pregnant women. While this study does not have direct impact on the pregnant women, the study results will help to provide better preterm birth management in the future. Pregnant women in emergency clinical settings will not be recruited.

Participants will undergo an additional procedure to quantify cervical stiffness. The study investigator will decide case by case if this additional procedure and the potential risk is acceptable for the pregnant women.

Each subject will undergo one speculum examination for checking the eligibility of the study, 3 consecutive TVUS cervical length and uterocervical angle assessments during two visits (it is an extra measurement only for the non-pessary cohort in the follow-up visit) and 3 consecutive cervical stiffness measurements during two visits.

Speculum examination and TVUS cervical length assessment are standard examinations for pregnant women and no additional risk is associated with this additional examination. The cervical stiffness measurement is done with the Pregnolia system. It has been CE-marked by TÜV Süd. Part of the registration process included a risk-benefit analysis of the Pregnolia system.

The Pregnolia System allows the assessment of cervical stiffness and its monitoring. The system provides an objective, more hygienic and less intrusive way of measuring cervical stiffness than cervical palpation. Possible side effects can be found in the Instruction for Use (IFU). However, the device seems to have a very good safety profile as the manufacturer received no serious adverse event reporting related to the medical device until now, neither in studies nor from the market.

The comparison of the benefits of the Pregnolia system with the risks shows that the benefit outweighs any of the potential risks considering their nature and frequency to prevent them.

### Study objectives

The primary objective is to determine the absolute value of CSI and its change over time in women undergoing pessary treatment, in comparison to normal pregnancies, when measured at 18^+0^ to 24^+6^ weeks and at the follow-up visit (4 weeks later).

The secondary objective is to determine the correlation of the initial CSI and CSI changes with birth outcome (gestational age at birth).

The safety objective is the safety of the device, by assessing incidence, severity, and seriousness of device-related adverse events.

### Study endpoints

The primary endpoint of the study is the Cervical Stiffness Index (CSI, in mbar). The secondary endpoint is patient delivery data (including gestational age, mode of delivery and complications). The safety endpoint is device-related adverse events (incidence, severity, and seriousness).

### Study assessments

The participation in the study will be added to the medical record. Additionally, the access to medical records is needed for the collection of study-relevant data such as previous history of the spontaneous PTB, demographic data, etc.

#### Assessment of primary endpoint

The primary endpoint is assessed via 3 consecutive CSI measurements with the Pregnolia System to obtain the CSI values. During all the visits the CSI measurements with the Pregnolia System are done according to the IFU. Briefly, the Pregnolia Control Unit is set up and one Pregnolia Probe, single use and sterile, is connected to the control unit. The woman is prepared for a speculum-based vaginal examination, excessive mucus is removed, and the measurement site is checked for contraindications / withdrawal criteria. The pump is started, and the probe is gently applied on the cervix at 12 o’clock position. The user is guided through the different steps of the measurement by audio signals. The CSI assessment is performed 3 consecutive times, and thereafter the probe is removed. The results (CSI1, CSI2 and CSI3) are reported in the CRF. The study team will be trained to ensure the right handling of the device.

The measurements should be made consecutively. In case of a measurement failure, this will be recorded in the CRF.

#### Assessment of secondary endpoint

Patient delivery data will be collected in the medical records of the subjects, within 4–8 weeks after birth / study completion. The following information will be collected: a collection of interventions and/or treatments during pregnancy to prevent preterm birth, if any, a collection of delivery information (onset of labour, mode of delivery, GA at delivery, date of delivery) and new-born data (e.g. weight, neonatal morbidities), a collection of health status data of mother (hospitalization (yes/no), indication for hospitalization, gestational age (GA) at admission and GA/date at discharge) and neonatal morbidities, a collection of health economics (date admission to NICU of the neonates, date of discharge from NICU, discharge from hospital).

#### Assessment of safety endpoints

To assess the safety of the Pregnolia system the occurrence of any serious adverse event (SAE) during the study will be rated according to their seriousness and relatedness.

All SAEs that occur during the study period (from time of consent to delivery), whether considered to be related to the study medical device or not, must be reported via the case report form. Additionally, all adverse device effects shall be reported in the CRF.

Infections will be collected as an adverse event but displayed additionally to the planned general overview of adverse events (AE).

#### Other assessments

The cervical length and the uterocervical angle will be measured with the use of a transvaginal ultrasound: three (3) consecutive TVUS scannings of the uterine and cervical anatomy will be taken according to fetal medicine procedure [23]. The patients will be in a dorsal lithotomy position with the bladder essentially empty. The pressure from the ultrasound probe on the cervix should be as gentle as possible [24].

**Fig 3:**
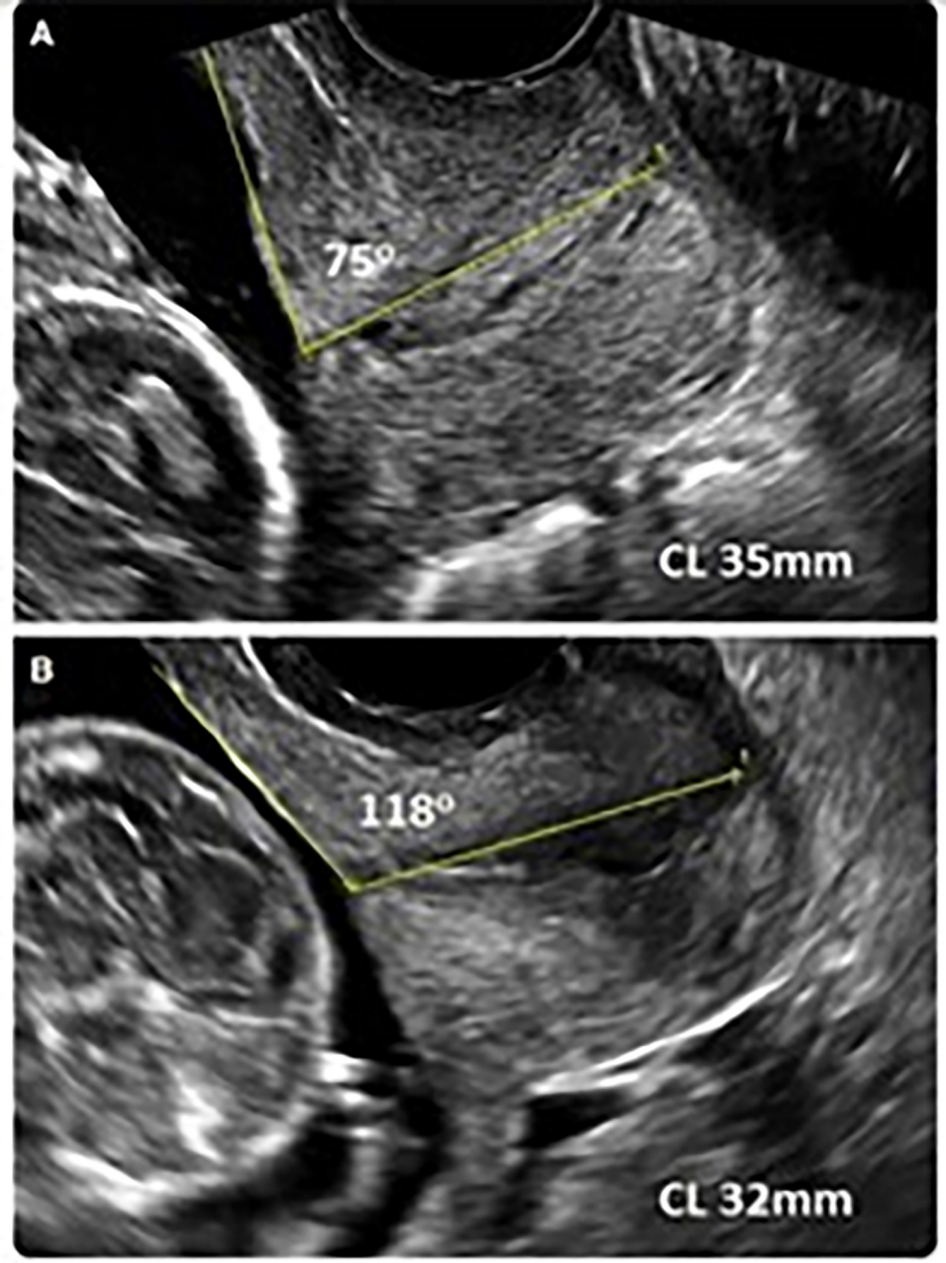
The measurement of UCA. From [25].

The cervix should be measured along its longitudinal axis. The UCA is the angle formed by the cervix and the lower uterine segment. The uterocervical angle (UCA) is measured on the image with the shortest cervix length (CL). First, a straight line is drawn between the external os and the internal os, including the isthmus. Second, a line parallel to the lower anterior uterine wall and passing through the end of the first line in the internal os is drawn (ideally 3 cm long). The angle given by the intersection of the two lines is measured and represents the UCA [25–27]. The ultrasound measurement of the UCA and CL is depicted in Fig 3.

### Data management and confidentiality

The investigators will use CRFs with all relevant data of the participating subjects. CRFs will be kept monthly updated to reflect the patient’s status at each phase during the study. The data will be pseudonymized and only research study staff will have access to the identification key. The medical record might be reviewed on site by a monitor for source data verification or any other quality check or during an audit, however all involved staff is obliged to maintain confidentiality.

#### Data collection and follow-up for withdrawn participants

A patient can be withdrawn from the study participation at any time. In case a subject is withdrawn from the study, no further data for that subject will be collected, but the data already collected will be used for analysis. Since there is no risk for the subject, no further follow-up will be required.

### Statistical analysis and methods

**Fig 4:**
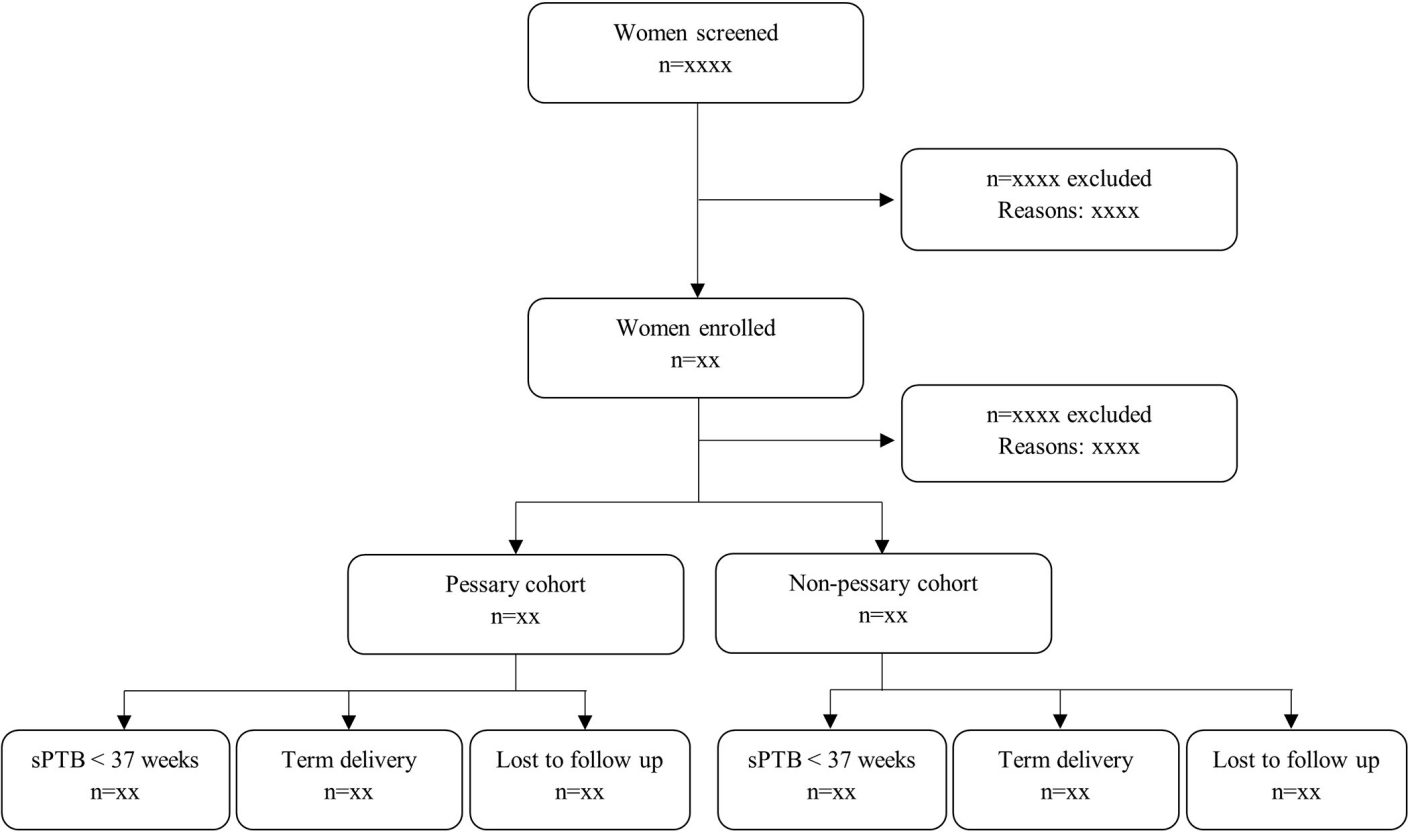
Flowchart summarizing the distribution of subjects in the study. sPTB = spontaneous preterm birth.

Distribution of subjects in the study will be presented as in the flowchart reported in Fig 4.

The CSI is a continuous variable. Consecutive CSI values (CSI 1, CSI 2 CSI 3) will be analysed and/or pooled as mean value, single sequential values, max or min value of the sequence, or combination of those and other possibilities.

Descriptive statistical methods will be used to summarize the data from this study. Unless stated otherwise, the term “descriptive statistics” refers to number of subjects (n), mean, median, standard deviation (SD), minimum, and maximum for continuous data and frequencies and percentages for categorical data. Multi-variate linear and logistic regression analysis will be performed, correlations and predictive capabilities for the CSI alone and in combination with other collected data will be evaluated. The level of significance will be 0.05.

The investigator will actively ask for any Adverse Events (AEs) during the visits. At each clinical investigation visit the investigator will assess and record any AE in detail in the source data. The Investigator will evaluate Serious Adverse Events (SAEs) regarding causality and seriousness. The number and percentage of participants with SAEs will be displayed by body system and preferred term using Medical Dictionary for Regulatory Activities (MedDRA) version 22.0 or higher, by cohort group and overall. Summaries in terms of severity and relationship will also be provided.

#### Monitoring

Monitoring at the investigator’s site during the study will be carried out to follow up the progress of the clinical investigation, to assure outmost accuracy of the data and to detect possible errors as early as possible. A Monitoring plan (according to ISO 14155 as far as applicable) pre-defines the amount of monitoring.

## Discussion

The study aims to evaluate whether patients indicated for pessary treatment have *a priori* a lower ectocervical stiffness than normal pregnancies. Moreover, this study aims to evaluate the impact of treatment with pessary on measured ectocervical stiffness, and whether it, or its changes correlate with birth outcome.

The uterine cervix plays a fundamental role in ensuring that a pregnancy is carried to term, while securing a closed uterus throughout gestation, it progressively softens, and finally opens to allow delivery. This constitutes a striking transformation of the tissue structure and characteristics, constituting a complex biomechanical process, part of a larger context of dynamic changes in structural forces, loads, deformations, and material and biochemical properties that occur during pregnancy. When a cervix is no longer structurally sound, it cannot perform its function adequately, which may lead to premature birth. Multiple aetiologies can lead to such a failure, but the overall knowledge of what in fact leads to cervical failure is far from complete.

The major parameter currently used to aid the identification of those women at risk of delivering preterm is the measurement of cervical length using transvaginal ultrasound. Cervical shortening is predictive of preterm birth, however cervical length has only limited predictive capacity [28, 29]. Understanding of cervical remodelling is fundamental for prediction of sPTB and to avoid pessary failure. The Pregnolia system is a device that performs a biomechanical function, being able to describe the biomechanical parameters of the cervix accurately and quantitatively.

With improved prediction of sPTB, asymptomatic women may benefit through early detection of lower or declining cervical stiffness values and receive pessary therapy even before cervical shortening [30].

This study investigates a fundamental biomechanical parameter of the cervix–likely connected to the competency of the cervix–in order to assess its contribution to an existing therapy. The benefit of this study could lead to better identification of patients that can or cannot benefit from the pessary therapy, and therefore lead to better preterm birth management.

Our study will lead to a better understanding of the impact of the use of pessary on ectocervical stiffness. We hypothesise that the CSI values may differ significantly across cohorts in the study. We are confident that the CSI in combination with TVUS, could be a potential diagnostic tool, which enables the identification of the group of pregnant women for the antenatal cervical pessary treatment.

## References

[1] Liu L, Oza S, Hogan D, Perin J, Rudan I, Lawn JE, et al. Global, regional, and national causes of child mortality in 2000–13, with projections to inform post-2015 priorities: an updated systematic analysis. Lancet. 2015;385(9966):430–40. Epub 2014/10/05. doi: 10.1016/S0140-6736(14)61698-6 . 25280870

[2] Ciancimino L, Lagana AS, Chiofalo B, Granese R, Grasso R, Triolo O. Would it be too late? A retrospective case-control analysis to evaluate maternal-fetal outcomes in advanced maternal age. Arch Gynecol Obstet. 2014;290(6):1109–14. Epub 2014/07/17. doi: 10.1007/s00404-014-3367-5 . 25027820

[3] Terzic M, Aimagambetova G, Terzic S, Radunovic M, Bapayeva G, Lagana AS. Periodontal Pathogens and Preterm Birth: Current Knowledge and Further Interventions. Pathogens. 2021;10(6). Epub 2021/07/03. doi: 10.3390/pathogens10060730 ; PubMed Central PMCID: PMC8227634. 34207831 PMC8227634

[4] Romero R, Mazor M, Munoz H, Gomez R, Galasso M, Sherer DM. The preterm labor syndrome. Ann N Y Acad Sci. 1994;734:414–29. Epub 1994/09/30. doi: 10.1111/j.1749-6632.1994.tb21771.x . 7978942

[5] Goldenberg RL, Culhane JF, Iams JD, Romero R. Epidemiology and causes of preterm birth. Lancet. 2008;371(9606):75–84. Epub 2008/01/08. doi: 10.1016/S0140-6736(08)60074-4 ; PubMed Central PMCID: PMC7134569. 18177778 PMC7134569

[6] Kyvernitakis I, Berger R, Maul H. Letter to the Editor: FIGO good practice recommendations on the use of pessary for reducing the frequency and improving outcomes of preterm birth. Int J Gynaecol Obstet. 2022;157(1):216–7. Epub 2022/02/08. doi: 10.1002/ijgo.14099 . 35128647

[7] Kyvernitakis I, Maul H, Rath W, Kraft K, Kuon R, Hamza A, et al. Position Paper of the Task Force for Obstetrics and Prenatal Medicine (AGG—Section Preterm Birth) on the Placement, Removal and Surveillance of the Arabin Cervical Pessary in Patients at Risk for Spontaneous Preterm Birth. Geburtshilfe Frauenheilkd. 2019;79(11):1171–5. Epub 2019/11/19. doi: 10.1055/a-1007-8613 ; PubMed Central PMCID: PMC6846727. 31736505 PMC6846727

[8] Goya M, de la Calle M, Pratcorona L, Merced C, Rodo C, Munoz B, et al. Cervical pessary to prevent preterm birth in women with twin gestation and sonographic short cervix: a multicenter randomized controlled trial (PECEP-Twins). Am J Obstet Gynecol. 2016;214(2):145–52. Epub 2015/12/03. doi: 10.1016/j.ajog.2015.11.012 . 26627728

[9] Goya M, Pratcorona L, Merced C, Rodo C, Valle L, Romero A, et al. Cervical pessary in pregnant women with a short cervix (PECEP): an open-label randomised controlled trial. Lancet. 2012;379(9828):1800–6. Epub 2012/04/06. doi: 10.1016/S0140-6736(12)60030-0 . 22475493

[10] Pratcorona L, Goya M, Merced C, Rodo C, Llurba E, Higueras T, et al. Cervical pessary to reduce preterm birth <34 weeks of gestation after an episode of preterm labor and a short cervix: a randomized controlled trial. Am J Obstet Gynecol. 2018;219(1):99 e1– e16. Epub 2018/04/29. doi: 10.1016/j.ajog.2018.04.031 . 29704487

[11] Saccone G, Maruotti GM, Giudicepietro A, Martinelli P, Italian Preterm Birth Prevention Working G. Effect of Cervical Pessary on Spontaneous Preterm Birth in Women With Singleton Pregnancies and Short Cervical Length: A Randomized Clinical Trial. JAMA. 2017;318(23):2317–24. Epub 2017/12/21. doi: 10.1001/jama.2017.18956 ; PubMed Central PMCID: PMC5820698 ICMJE Form for Disclosure of Potential Conflicts of Interest and none were reported. 29260226 PMC5820698

[12] van ’t Hooft J, van der Lee JH, Opmeer BC, van Wassenaer-Leemhuis AG, van Baar AL, Bekedam DJ, et al. Pessary for prevention of preterm birth in twin pregnancy with short cervix: 3-year follow-up study. Ultrasound Obstet Gynecol. 2018;51(5):621–8. Epub 2018/02/23. doi: 10.1002/uog.19029 . 29468770

[13] Dang VQ, Nguyen LK, Pham TD, He YTN, Vu KN, Phan MTN, et al. Pessary Compared With Vaginal Progesterone for the Prevention of Preterm Birth in Women With Twin Pregnancies and Cervical Length Less Than 38 mm: A Randomized Controlled Trial. Obstet Gynecol. 2019;133(3):459–67. Epub 2019/02/12. doi: 10.1097/AOG.0000000000003136 . 30741812

[14] Cannie MM, Dobrescu O, Gucciardo L, Strizek B, Ziane S, Sakkas E, et al. Arabin cervical pessary in women at high risk of preterm birth: a magnetic resonance imaging observational follow-up study. Ultrasound Obstet Gynecol. 2013;42(4):426–33. Epub 2013/05/15. doi: 10.1002/uog.12507 . 23671013

[15] Fernandez M, House M, Jambawalikar S, Zork N, Vink J, Wapner R, et al. Investigating the mechanical function of the cervix during pregnancy using finite element models derived from high-resolution 3D MRI. Comput Methods Biomech Biomed Engin. 2016;19(4):404–17. Epub 2015/05/15. doi: 10.1080/10255842.2015.1033163 ; PubMed Central PMCID: PMC4644115. 25970655 PMC4644115

[16] Arabin B, Alfirevic Z. Cervical pessaries for prevention of spontaneous preterm birth: past, present and future. Ultrasound Obstet Gynecol. 2013;42(4):390–9. Epub 2013/06/19. doi: 10.1002/uog.12540 ; PubMed Central PMCID: PMC4282542. 23775862 PMC4282542

[17] Kyvernitakis I, Maul H, Bahlmann F. Controversies about the Secondary Prevention of Spontaneous Preterm Birth. Geburtshilfe Frauenheilkd. 2018;78(6):585–95. Epub 2018/07/03. doi: 10.1055/a-0611-5337 ; PubMed Central PMCID: PMC6018068. 29962517 PMC6018068

[18] Owen J, Yost N, Berghella V, MacPherson C, Swain M, Dildy GA 3rd, et al. Can shortened midtrimester cervical length predict very early spontaneous preterm birth? Am J Obstet Gynecol. 2004;191(1):298–303. Epub 2004/08/06. doi: 10.1016/j.ajog.2003.11.025 . 15295382

[19] Newman RB, Goldenberg RL, Iams JD, Meis PJ, Mercer BM, Moawad AH, et al. Preterm prediction study: comparison of the cervical score and Bishop score for prediction of spontaneous preterm delivery. Obstet Gynecol. 2008;112(3):508–15. Epub 2008/09/02. doi: 10.1097/AOG.0b013e3181842087 ; PubMed Central PMCID: PMC2728002. 18757646 PMC2728002

[20] Badir S, Bernardi L, Feijo Delgado F, Quack Loetscher K, Hebisch G, Hoesli I. Aspiration technique-based device is more reliable in cervical stiffness assessment than digital palpation. BMC Pregnancy Childbirth. 2020;20(1):391. Epub 2020/07/08. doi: 10.1186/s12884-020-03080-x ; PubMed Central PMCID: PMC7339509. 32631265 PMC7339509

[21] Wang B, Zhang Y, Chen S, Xiang X, Wen J, Yi M, et al. Diagnostic accuracy of cervical elastography in predicting preterm delivery: A systematic review and meta-analysis. Medicine (Baltimore). 2019;98(29):e16449. Epub 2019/07/25. doi: 10.1097/MD.0000000000016449 ; PubMed Central PMCID: PMC6708731. 31335700 PMC6708731

[22] Badir S, Mazza E, Zimmermann R, Bajka M. Cervical softening occurs early in pregnancy: characterization of cervical stiffness in 100 healthy women using the aspiration technique. Prenat Diagn. 2013;33(8):737–41. Epub 2013/04/05. doi: 10.1002/pd.4116 . 23553612

[23] Clement S, Candy B, Heath V, To M, Nicolaides KH. Transvaginal ultrasound in pregnancy: its acceptability to women and maternal psychological morbidity. Ultrasound Obstet Gynecol. 2003;22(5):508–14. Epub 2003/11/18. doi: 10.1002/uog.893 . 14618665

[24] Kagan KO, Sonek J. How to measure cervical length. Ultrasound Obstet Gynecol. 2015;45(3):358–62. Epub 2015/01/30. doi: 10.1002/uog.14742 . 25632014

[25] Dziadosz M, Bennett TA, Dolin C, West Honart A, Pham A, Lee SS, et al. Uterocervical angle: a novel ultrasound screening tool to predict spontaneous preterm birth. Am J Obstet Gynecol. 2016;215(3):376 e1-7. Epub 2016/03/29. doi: 10.1016/j.ajog.2016.03.033 . 27018466

[26] Farras Llobet A, Higueras T, Calero IZ, Regincos Marti L, Maiz N, Goya MM, et al. Prospective evaluation of the uterocervical angle as a predictor of spontaneous preterm birth. Acta Obstet Gynecol Scand. 2020;99(11):1511–8. Epub 2020/04/21. doi: 10.1111/aogs.13879 . 32311754

[27] Luechathananon S, Songthamwat M, Chaiyarach S. Uterocervical Angle and Cervical Length as a Tool to Predict Preterm Birth in Threatened Preterm Labor. Int J Womens Health. 2021;13:153–9. Epub 2021/02/12. doi: 10.2147/IJWH.S283132 ; PubMed Central PMCID: PMC7868249. 33568951 PMC7868249

[28] Hughes K, Kane SC, Araujo Junior E, Da Silva Costa F, Sheehan PM. Cervical length as a predictor for spontaneous preterm birth in high-risk singleton pregnancy: current knowledge. Ultrasound Obstet Gynecol. 2016;48(1):7–15. Epub 2015/11/12. doi: 10.1002/uog.15781 . 26556674

[29] Ferrero DM, Larson J, Jacobsson B, Di Renzo GC, Norman JE, Martin JN Jr., et al. Cross-Country Individual Participant Analysis of 4.1 Million Singleton Births in 5 Countries with Very High Human Development Index Confirms Known Associations but Provides No Biologic Explanation for 2/3 of All Preterm Births. PLoS One. 2016;11(9):e0162506. Epub 2016/09/14. doi: 10.1371/journal.pone.0162506 ; PubMed Central PMCID: PMC5021369 global health issues. Authors from BCG (DMF, JL, SCS) were employed under contract with the March of Dimes Foundation and FIGO. This does not alter our adherence to PLOS ONE policies on sharing data and materials. 27622562 PMC5021369

[30] Blackwell SC, Sullivan EM, Petrilla AA, Shen X, Troeger KA, Byrne JD. Utilization of fetal fibronectin testing and pregnancy outcomes among women with symptoms of preterm labor. Clinicoecon Outcomes Res. 2017;9:585–94. Epub 2017/10/19. doi: 10.2147/CEOR.S141061 ; PubMed Central PMCID: PMC5633307. 29042802 PMC5633307

